# Cardiovascular Magnetic Resonance Imaging in the Early Detection of Cardiotoxicity Induced by Cancer Therapies

**DOI:** 10.3390/diagnostics12081846

**Published:** 2022-07-30

**Authors:** Xiaoting Wei, Ling Lin, Guizhi Zhang, Xuhui Zhou

**Affiliations:** Department of Radiology, The Eighth Affiliated Hospital of Sun Yat-sen University, Shenzhen 518036, China; weixting2020@126.com (X.W.); linling26@mail.sysu.edu.cn (L.L.)

**Keywords:** cardiotoxicity, cardiovascular magnetic resonance, mapping, strain

## Abstract

The significant progress in cancer treatment, including chemotherapy, immunotherapy, radiotherapy, and combination therapies, has led to higher long-term survival rates in cancer patients, while the cardiotoxicity caused by cancer treatment has become increasingly prominent. Cardiovascular magnetic resonance (CMR) is a non-invasive comprehensive imaging modality that provides not only anatomical information, but also tissue characteristics and cardiometabolic and energetic assessment, leading to its increased use in the early identification of cardiotoxicity, and is of major importance in improving the survival rate of cancer patients. This review focused on CMR techniques, including myocardial strain analysis, T1 mapping, T2 mapping, and extracellular volume fraction (ECV) calculation in the detection of early myocardial injury induced by cancer therapies. We summarized the existing studies and ongoing clinical trials using CMR for the assessment of subclinical ventricular dysfunction and myocardial changes at the tissue level. The main focus was to explore the potential of clinical and preclinical CMR techniques for continuous non-invasive monitoring of myocardial toxicity associated with cancer therapy.

## 1. Introduction

The significant progress in cancer treatment has led to higher long-term survival rates in cancer patients [1,2], with more than 16.9 million cancer survivors in the United States in 2019, and is expected to reach 22.1 million by 2030 [3]. At the same time, adverse effects induced by chemotherapy, immunotherapy, radiotherapy, targeted therapy, and combination therapies have become increasingly prominent. Cardiotoxicity, which was identified in the 1970s in patients receiving anthracyclines, has been one of the most serious complications in cancer therapy [4,5,6]. The incidence of chemotherapy-agent-related cardiotoxicity ranges from 0.2% to 48% [7]. Other cancer therapies, such as radiotherapy [8], immunotherapy, targeted therapy [9], and/or combinations of multiple therapies, are also associated with cardiovascular toxic effects. Cardiotoxicity induced by cancer therapies includes structural and functional changes in the myocardium, pericardium, coronary artery, valves, and large vessels, and ultimately may lead to major adverse cardiac events (MACEs) [7].

The clinical diagnosis of cardiotoxicity is mainly based on abnormal left ventricular ejection fraction (LVEF) and the presence of heart failure symptoms [10]. In recent years, the diagnostic criteria of cardiotoxicity were proposed by several academic societies, including the American Society of Echocardiography (ASE), the European Society of Cardiology (ESC), the European Society for Medical Oncology (ESMO), and the International Cardio-Oncology Society (IC-OS) (Table 1). However, an international unified consensus on the criteria, as well as the best modality and/or biomarkers, has not been reached, especially for asymptomatic patients. Electrocardiograms, cardiac biomarkers, echocardiography, multi-gated radionuclide angiography (MUGA), and cardiovascular magnetic resonance (CMR) have been used to detect cardiotoxicity. Endomyocardial biopsy (EMB) was historically used to monitor the extent of anthracycline-related cardiotoxicity based on histological changes [11], but is now less performed due to its limitations, such as invasivity, the chance of sampling errors, and the risk of severe complications [12]. There has been an increasing need to assess the functional changes and myocardial injury in cardiotoxicity in a non-invasive, safe and accurate manner. The following principles are suggested when choosing diagnostic modalities: (1) selecting the same modality and/or biomarker with high sensitivity and reproducibility throughout the treatment pathway; (2) providing as much relevant clinical information as possible, including right ventricular (RV) function, valvular function, pericardium assessment, etc.; (3) no radiation exposure if available; and (4) LV global longitudinal strain (GLS) may be considered [7,13].

Studies have shown that approximately 58–89% of patients do not fully recover from LVEF after the treatment for cardiotoxicity caused by LVEF [16,17,18,19]. Early detection of cardiotoxicity and prompt initiation of cardioprotective therapy may help to reverse its course. Moreover, it is crucial to modify chemotherapy regimens, including the administration method, dosage form (liposomal encapsulation), and dose, or even to cease chemotherapy when necessary, in order to restore LV function and reduce adverse cardiac events [20,21,22]. These findings have led to an increased interest in the early detection of subclinical cardiotoxicity, and have promoted the establishment and rapid development of the cardio-oncology specialty.

CMR is a non-invasive comprehensive imaging modality that provides not only anatomical information, but also tissue characterization, as well as cardiometabolic and energetic assessment. This review focuses on the subclinical ventricular dysfunction and the myocardial changes at the tissue level assessed using CMR in an attempt to summarize the potential imaging biomarkers for the early diagnose of cardiotoxicity associated with cancer therapies. In particular, we highlighted the latest CMR studies investigating the right ventricle, which might be more susceptible to cardiotoxicity than the left ventricle, but has been less evaluated. Moreover, we checked the ongoing registered clinical trials using CMR techniques for the evaluation of cardiotoxicity, and proposed imaging strategies that can be incorporated in future clinical trials with different purposes.

## 2. Clinical Types and Pathophysiology of Cardiotoxicity

Cancer therapies can damage both tumor cells and normal body cells. As myocardial cells are terminally differentiated cells with a limited dividing ability, they are prone to long-term and serious results after being damaged. Based on the presence of structural abnormalities and the degree of functional reversibility, cardiotoxicity can be classified into two types: type I (the injury type) and type II (the dysfunction type) [23].

Type I cardiotoxicity refers to severe myocardium injury that is generally considered irreversible due to the structural damage [23]. The main representative agent that induces type I cardiotoxicity is anthracycline. Anthracycline is one of the most widely used chemotherapeutic agents; it is characterized by its broad spectrum and strong efficacy, and is the major cornerstone drug for the treatment of various solid tumors and hematological malignancies [24].However, anthracycline is known to cause cardiotoxicity, with incidence ranging from 6% to 18% [17,25]. Anthracycline-induced cardiotoxicity is dose-dependent, progressive, cumulative, and leads to irreversible myocardial damage. However, treatment with a low dose of anthracycline can also lead to potential myocardial damage and late complications [26]. Several known risk factors for anthracycline-induced cardiotoxicity include age, being female, hypertension, and mediastinal radiotherapy [17,27,28].

In the past three decades, the pathogenesis of anthracycline-induced cardiotoxicity has been studied intensively, but the exact mechanism is not entirely clear. The most widely accepted mechanism is that topoisomerase II beta and reactive oxygen species (ROS) play the key roles [29]. Anthracycline destroys the double-helical structure of deoxyribonucleic acid (DNA) by blocking the topoisomerase II beta and inhibits the synthesis and mitochondrial biogenesis of DNA and ribonucleic acid (RNA) [30]. In addition to the anti-cancer effect, the production of ROS increases under anthracycline therapy, which can impact cardiac morphology and function [31,32]. The mechanisms are as follows: (1) increased ROS may affect cardiac contraction by altering the activity of the sarcoplasmic reticulum Ca^+2^ release channel and the ryanodine receptor; (2) ROS can mediate myocardial cell apoptosis by activating hypertrophy signal kinases and transcription factors; and (3) excessive ROS stimulate cardiac fibroblast proliferation and activate matrix metalloproteinases, leading to fibrosis and matrix remodeling [33,34]. The polyphenols, a group of bioactive compounds showing pleiotropic effects on the cardiovascular system, including an antioxidant effect, have been associated with improved prognosis of cardiovascular diseases [35]. The cardioprotective effect of grape polyphenol concentrate was observed in experimental rats with doxorubicin-induced cardiotoxicity, which also underlined the role of ROS in cardiotoxicity [36].

**Figure 1:**
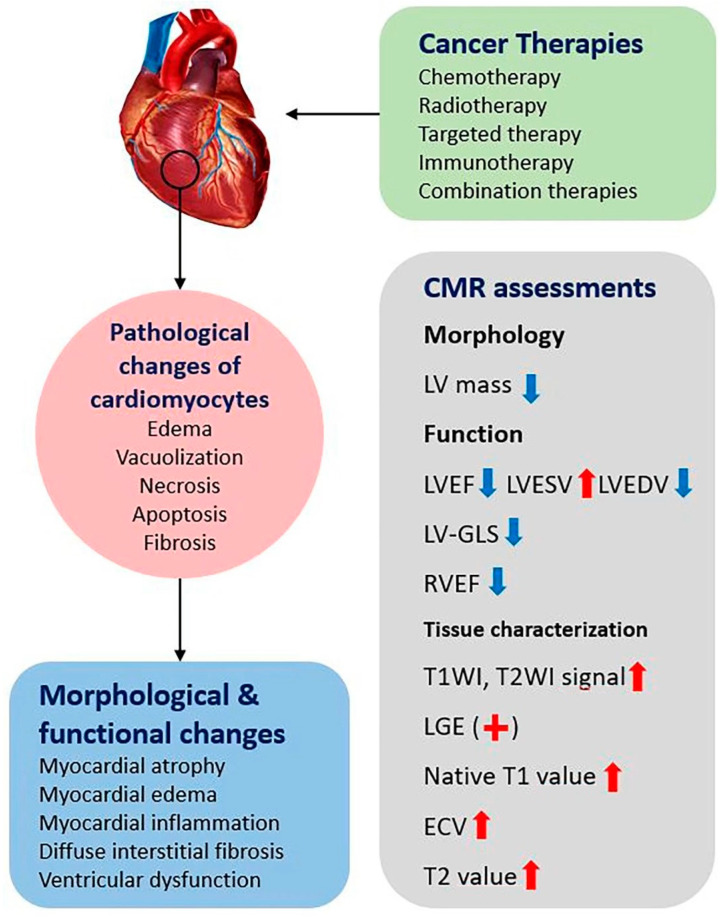
Overview of the pathological changes of cardiomyocytes in cardiotoxicity induced by cancer therapies, the resulting morphological and functional changes of the heart, and CMR assessments that have been performed in previous studies. Different CMR parameters may increase (red arrows) or decrease (blue arrows) or be positive (red plus) in cardiotoxicity. LV = left ventricular; LVEF = left ventricular ejection fraction; LVESV = LV end-systolic volume; LVEDV = LV end-diastolic volume; GLS = global longitudinal strain; RVEF = right ventricular ejection fraction; T1WI = T1-weighted imaging; T2WI = T2-weighted imaging; LGE = late gadolinium enhancement; ECV = extracellular volume fraction.

Pathological manifestations of cardiotoxicity include myocardial edema, cytoplasmic vacuolization, necrosis, apoptosis, and eventually myocardial and extracellular fibrosis (Figure 1) [37,38,39]. A prolonged T2 relaxation time or an increased gadolinium signal intensity in CMR resulted from vacuolization of myocardial cells, and myocardial edema were found in animal studies after the administration of doxorubicin [40,41]. Similar CMR manifestations were also observed in patients receiving chemotherapy at an early stage (≤3 months of chemotherapy), and myocardial fibrosis was found at a late stage (>12 months) [42].

Type II cardiotoxicity, which was defined in the early 2000s, is not dose-dependent and is highly reversible due to the absence of ultrastructural abnormalities [23]. The characteristic agent associated with type II cardiotoxicity is trastuzumab, which is a monoclonal antibody targeting the molecule HER-2. The pathophysiological change of the trastuzumab-induced cardiotoxicity is mainly attributed to the blocking of the ErbB2 signaling, which is required for the growth, repair, and survival of cardiomyocytes. However, anthracycline and trastuzumab are often applied sequentially or concurrently in cancer treatment, and may affect cardiac reserve and increase susceptibility to myocardial injury [14].

## 3. CMR Imaging Biomarkers in the Assessment of Cardiotoxicity

CMR is the non-invasive gold-standard imaging modality to assess the structural and functional changes of the heart by means of a variety of sequences. The changes in ventricular morphology and function, valvular function, the pericardium, and large vessels can be evaluated using dark-blood sequences, cine sequence, and phase-contrast flow sequences. Cine sequence can also be used to quantify functional parameters, such as LVEF, and to identify wall motion abnormalities. Myocardial tissue characterization techniques, such as gadolinium enhancement sequences and mapping techniques, enable the detection of myocardial edema, inflammation, and fibrosis.

### 3.1. Left Ventricular Morphology and Function

Conventional left ventricular parameters such as LV mass and LVEF have been essential for the diagnosis and prognosis of cardiotoxicity. Myocardial strain analysis offers the opportunity to detect subclinical dysfunction when LVEF remains normal.

#### 3.1.1. LV Mass

LV mass has attracted attention in cardio-oncology as a quantifiable marker for cardiotoxicity. LV mass reduction may represent myocardium atrophy, and the loss in LV cardiomyocytes was associated with the increase in peak cardiac troponin T after anthracycline treatment in a previous study [43]. CMR can be used to quantify LV mass accurately with high reproducibility [44]. Another study of 91 patients revealed a negative correlation between the index LV mass and anthracycline dose [45]. Furthermore, multivariable regression models showed that the index LV mass was the strongest predictor of subsequent MACE in comparison with the anthracycline dose, glomerular filtration rate, and CMR-derived LVEF [45].

#### 3.1.2. LVEF

LVEF was evaluated using echocardiography and MUGA in early studies of cardiotoxicity. Echocardiography is the first-line imaging modality to assess LVEF in clinical practice. However, the reproducibility can be impacted by its high operator dependency, and the accuracy may be challenged in patients with an inadequate acoustic window, such as breast cancer patients with breast expanders, prostheses, or implants [46]. The variability of two-dimensional echocardiography (2DE) LVEF can reach 11.5% to 26%, according to previous studies [44,47]. Another study showed that the mean LVEF in adult survivors of childhood cancer was overestimated by 5% by 2DE compared to CMR, and 11% of patients were misclassified as LVEF ≥ 50% by 2DE, resulting in false-negative diagnoses [48]. MUGA was also widely used in early studies to evaluate LVEF [49,50,51]. However, Huang et al. showed in a study of 75 patients that the use of MUGA-LVEF resulted in the misclassification of cardiotoxicity in up to 35% of patients [52]. In addition, MUGA is rarely used for follow-up surveillance of LVEF due to ionizing radiation exposure, an inability to provide morphological and functional information, and an inability to assess pericardial and valvular diseases [53]. A cross-sectional study showed that the usage of MUGA decreased from 30.4% to 16.7% (from 2011 to 2014), and CMR usage increased from 0.9% to 2.9% [54].

CMR can be used to monitor the changes in LVEF with excellent accuracy and reproducibility due to its high spatial resolution [44,55,56], and has been recommended for baseline evaluation in patients with poor echocardiographic image quality. A decrease in LVEF can be caused by a decrease in LV end-diastolic volume (LVEDV) or an increase in LV end-systolic volume (LVESV), or both. Previous studies suggested that an increased LVESV was the primary driver of reduced LVEF in patients treated with anthracycline and/or trastuzumab [57,58], which may have been triggered externally by an increased LV afterload or internally by myocardial contractile dysfunction [59]. Drafts et al. found that LVEF decreased with an increased LVESV in 53 patients within 6 months after low-to-moderate doses of anthracycline-based chemotherapy, and the decrease in LVEF was paralleled by the impaired LV mean mid-wall circumferential strain, suggesting that the reduction in myocardial contractility promoted by chemotherapy was the reason for the decrease in LVEF [58]. However, in a study of 120 patients who underwent chemotherapy, 19% of the decline in LVEF was due to an isolated decrease in LVEDV, which was caused by volume depletion (diminished LV preload) [60]. Intravascular hypovolemia as a result of poor oral intake, vomiting, or diarrhea during cancer treatment may lead to a decrease in LVEDV and LVEF, which may lead to incorrect implication of cardiotoxicity and erroneous termination of cancer treatment.

In addition, LVEF is insensitive to subtle myocardial injury or subclinical reduce of LV function [61,62]. Ewer et al. found that the correlation between LVEF and cardiac biopsy grade of cardiotoxicity was poor, and 6% of the patients with a biopsy grade greater than 2 (using a scale of 0–3) did not show a significant decrease in LVEF [63]. These findings indicate that the deterioration of LVEF might be a late manifestation that does not show until the myocardium has been severely damaged and has entered an irreversible stage. Therefore, the efficacy of LVEF in early detection of cardiotoxicity remains controversial, and biomarkers with greater sensitivity are urgently needed for the early identification of cardiotoxicity.

#### 3.1.3. Left Ventricular Strain

Strain refers to the ability of the myocardium to deform. LV strain depicts the LV myocardial deformation in a cardiac cycle as a percentage of its initial length. Measurements of the segmental and global strain of the myocardium in longitudinal, radial, and circumferential directions can partly reflect the internal contractile function of the myocardium [64]. Several studies have shown that myocardial strain correlates with LVEF in patients undergoing potentially cardio-toxic chemotherapy, and often precedes significant changes in LVEF [65,66,67]. The underlying mechanism might be as follows: (1) segmental changes in myocardial function may occur before the overall decrease in LV function, as the adjacent normal myocardium may help to maintain a normal LVEF through a compensatory mechanism in the early stage of myocardial dysfunction [68]; and (2) LVEF is not only related to myocardial strain, but also affected by end-diastolic wall thickness [69].

GLS is the changes in myocardial length from base to apex in a cardiac cycle, and is highly sensitive to subclinical LV dysfunction and has been included in an expert consensus for imaging evaluation of cardiotoxicity [14]. The consensus recommends that a decrease of >15% from the baseline in GLS is abnormal [14]. In a prospective study of 81 patients receiving trastuzumab, GLS was the best independent predictor for early cardiotoxicity and LVEF reduction [70]. A multicenter prospective randomized clinical trial showed that the initiation of cardioprotective therapy guided by GLS reduction ≥ 12% resulted in a smaller reduction in LVEF during follow-up compared with that guided by a LVEF reduction > 10%, supporting the value of GLS in surveillance for cardiotoxicity [71]. In addition, pre- and post-chemotherapy GLS values were independently associated with the development of MACE [72,73].

Circumferential strain reflects the degree of circumferential shortening of the ventricular myocardial fibers. Narayan et al. found that circumferential strain was one of the strongest predictive parameter of cardiotoxicity, with a 1% difference in the baseline circumferential strain associated with a 38% increase in the odds of cardiotoxicity [74]. Other studies have shown that LV mean mid-wall circumferential strain is associated with a subclinical decline in LVEF [58,75]. Drafts et al. found that LV mean mid-wall circumferential strain decreased within 6 months after anthracycline administration, corresponding to a reduction in LVEF [58].

While myocardium strain is garnering increasing attention, its limitations cannot be ignored. First of all, high inter-observer and intra-observer variability of strain parameters, especially that derived using echocardiography, is a substantial barrier to an extensive clinical application. Moreover, the need for dedicated post-processing consumes additional time and costs. Last but not least, various vendors and post-processing algorithms make it difficult to establish a universal reference range of normal values. A comparative study showed that the absolute difference of GLS among different vendors was up to 3.7% strain units [76]. Further standardization is needed before strain can be widely used in clinical settings.

#### 3.1.4. Right Ventricular Function

RV and biventricular impairment have also been found in cardiotoxicity, in addition to the primary concern for LV dysfunction. A recent study of cardiotoxicity in rats confirmed that histopathological changes occurring in the LV could also be detected in the right ventricle, even with more prevalent interstitial fibrosis [39]. Another study identified RV dysfunction in 34% of the patients (defined by a 10% drop in CMR-RVEF) at 12 months, which correlated with early myocardial edema [77]. A study of 62 long-term cancer survivors (median follow-up for 7.8 years) showed that 18% of the subjects demonstrated abnormal LVEF and 27% demonstrated abnormal RV function quantified using CMR [78]. It is noteworthy that in both studies, the incidence of RV dysfunction was higher than LV dysfunction, which indicated that the right ventricle might be more susceptible to cardiotoxicity, probably due to its thinner ventricular wall with fewer myofibrils. However, one study of lymphoma survivors showed that the proportion of survivors with LV dysfunction was higher than that of survivors with RV dysfunction, which contradicted the above studies [79]. RV dimensions and systolic parameters were evaluated by echocardiography and pulsed Doppler tissue imaging in this study. There are two plausible reasons for the different results: (1) the diagnosis of RV dysfunction was based on echocardiography-derived RV systolic parameters in this study, while CMR-derived RVEF was applied in other studies; and (2) echocardiography has well-recognized limitations in the evaluation of the right ventricle due to the irregular structure and less optimal acoustic window of the right ventricle compared with the left ventricle.

RV dysfunction is a reliable prognostic marker for adverse outcomes in a variety of cardiovascular diseases. It has been found that patients treated with trastuzumab who demonstrated biventricular dysfunctions had less improvement in LVEF than those without RV dysfunction, as assessed using echocardiography-derived LVEF, RV fractional area change, and peak systolic longitudinal strain (LV and RV) [80]. Further large-scale studies are required to evaluated the prognostic value of RV dysfunction in cardiotoxicity

RV strain can also be used to evaluate the subclinical dysfunction of the right ventricle. However, the feasibility of RV strain is limited by the thinner ventricular wall and the difficulty in tracking the endocardial boundaries. Only a few studies have analyzed RV strain using echocardiography and demonstrated the value of RV strain in the early detection and prognosis of cardiotoxicity [79,80,81]. CMR might improve the reproducibility and accuracy of RV strain assessment, and thus deserves further study.

### 3.2. Myocardial Tissue Characterization

Tissue characterization techniques in CMR enable the detection of microstructural changes in the myocardium in the process of a variety of myocardial diseases, and have been widely used in the diagnosis and follow-up of cardiomyopathy, myocardial infiltration, and inflammatory diseases [82].

Conventional sequences, including T1-weighted, T2-weighted, and cine bright-blood steady-state free-precession sequences, can provide basic morphological information on the myocardium, as well as the surrounding structures (pericardium and valves), and extra-cardiac information (pleural effusion and metastases). An early animal study showed that an increase in the myocardial T1 signal intensity may be related to subclinical injury caused by oxidative stress [83]. CMR is the primary imaging modality for the assessment of myocardial edema and inflammation. The most common approach is a semi-quantitative detection of edematous tissue using T2-weighted imaging, which indicates myocardial edema when a high signal intensity or a ratio of the myocardial to skeletal muscle signal intensity greater than 1.9 is observed [84,85]. A study of 46 breast cancer patients treated with potentially cardio-toxic chemotherapy showed an increase in the ratio of the myocardial to skeletal muscle signal intensity 1 month after chemotherapy, which was associated with a decreased RVEF at 12 months [77]. However, conventional T1- and T2-weighted imaging is insensitive to mild microstructural changes, and conventional T2-weighted imaging may fail to recognize global myocardial edema.

The late gadolinium enhancement (LGE) technique is considered pivotal for tissue characterization and can identify regional myocardial fibrosis, providing prognostic information for a variety of cardiovascular diseases [38,86,87,88]. An increased gadolinium signal intensity was found in doxorubicin-toxic rat models with histopathologic evidence of myocardial vacuolization, and predicted the deterioration of LVEF [41]. In addition, another study of 10 patients with non-Hodgkin lymphoma treated with doxorubicin found that three (30%) patients had a segment of LGE at 3 months after chemotherapy [89]. However, most patients diagnosed with cardiotoxicity did not have myocardial LGE during or after chemotherapy in clinical studies [58,65,90,91]. It was considered that the pathological manifestation of cardiotoxicity is diffuse interstitial fibrosis, rather than focal fibrosis, and that LGE cannot be used to identify abnormalities in patients with diffuse myocardial fibrosis, in view there being no normal myocardial comparison. A longitudinal study found that myocardial T1 values measured on LGE images increased in all segments after chemotherapy, and were significantly correlated with pre-contrast T1 values, with a trend of correlation with the decline in LVEF [57].

**Figure 2:**
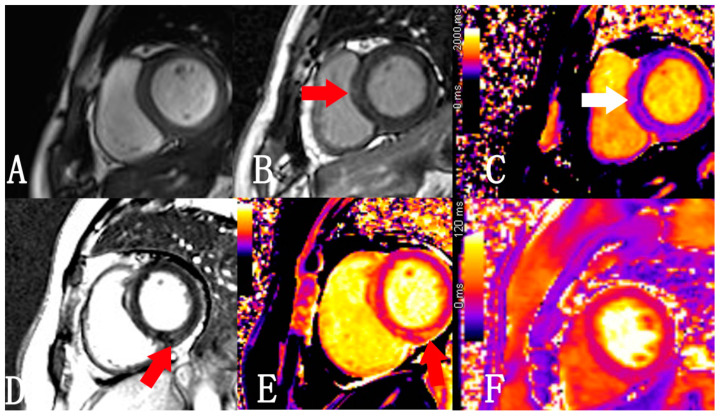
(**A**–**C**) A 61-year-old man with multiple myeloma who had undergone 4 cycles of chemotherapy within 5 months (bortezomib 2.25 mg, lenalidomide 25 mg, and dexamethasone 40 mg). (**A**) LVEF calculated from cine images was normal (68.2%). (**B**) LGE image showing an intramural, slightly hyperintense signal in the ventricular septum (arrow). (**C**) Native T1 mapping showing a larger T1 value for the ventricular septum than that of the lateral wall (1274 ms and 1173 ms, respectively). (**D**,**E**) A 62-year-old woman with multiple myeloma for 6 years. The chemotherapy regimen included 5 cycles of CTD (cyclophosphamide 1.2 g, thalidomide 200 mg, and dexamethasone 20 mg), 2 cycles of VTD (bortezomib 2.3 mg, thalidomide 100 mg, and dexamethasone 17 mg), and 2 cycles of VRD (bortezomib 2.3 mg, lenalidomide 25 mg, and dexamethasone 17 mg). The electrocardiogram showed a prolonged QT interval. (**D**) LGE image demonstrating a slightly hyperintense lesion in the LV inferior wall (arrow). (**E**) Native T1 mapping showing a significantly larger T1 value for the lesion compared to that of lateral wall (1568 ms and 1232 ms, respectively). (**F**) No significant increase in the T2 value was found in the T2 mapping, suggesting no significant edema (41 ms and 37 ms, respectively). LVEF = left ventricular ejection fraction; LGE = late gadolinium enhancement.

Parametric mapping is an innovative imaging technique that can achieve higher-level tissue features by directly quantifying pixels without reference to putative normal tissue. Parametric mapping techniques, including T1 mapping, T2 mapping, and extracellular volume fraction (ECV), can overcome the limitations of conventional T1-weighted imaging, T2-weighted imaging, and LGE in assessing diffuse myocardial inflammation or fibrosis [92,93,94,95]. T1 mapping and T2 mapping can improve the objectivity and can detect microstructural changes in the myocardium before functional changes are detected, which is expected to become the standard for comprehensive non-invasive myocardial tissue characterization in the future (Figure 2).

**Figure 3:**
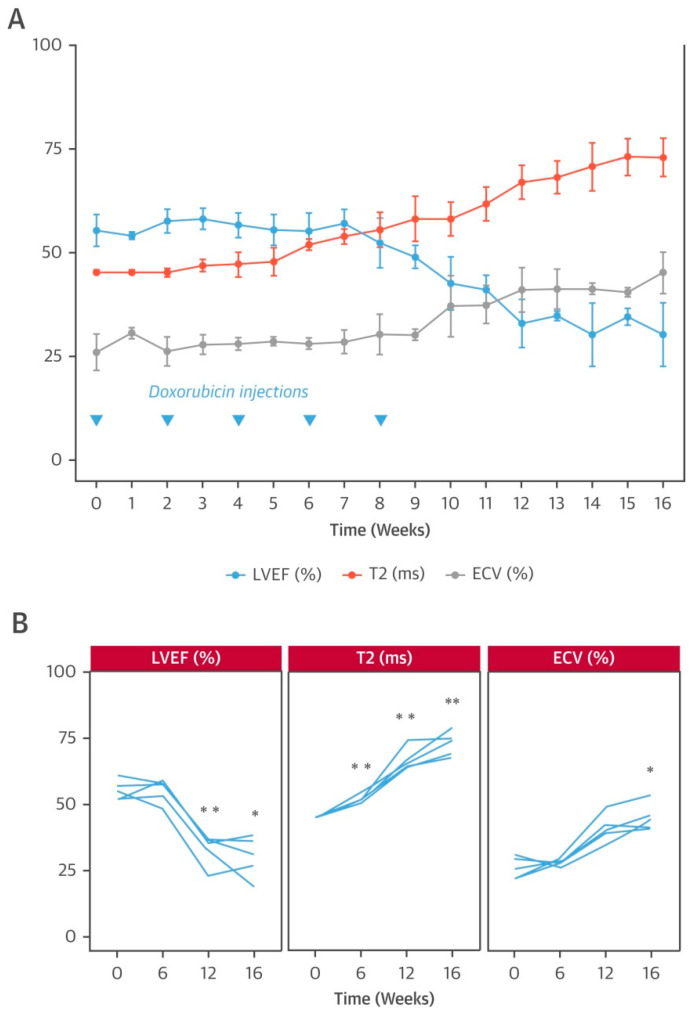
Animal model of anthracycline cardiotoxicity. (**A**) Time course of CMR imaging studies in pig model that received 5 biweekly doxorubicin injections and follow-up to week 16. Data are represented as mean ± SD (bars); (**B**) Individual animal data at selected time points for LVEF, T2, and ECV. Asterisks indicate statistically significant differences compared with week 0: * *p* < 0.05, ** *p* < 0.01. Reprinted with permission from Ref. [40]. 2019, Elsevier.

T1/T2 mapping has been useful in the diagnosis of myocardial edema and in ruling out active inflammation via quantification of T1/T2 relaxation time, especially T2 relaxation [96,97,98], demonstrating a good agreement with microscopic changes [99]. A study in swine models found that T2 relaxation time was prolonged early (at week 6) after intra-coronary doxorubicin injection due to the increased myocardial water content, while the T1 relaxation time and ECV were unaffected at this stage, in concordance with the pathological finding of intracellular edema without extracellular space expansion. Therefore, an abnormal T2 relaxation time may serve as an early marker of cardiotoxicity, reflecting myocardial injury at a reversible stage (Figure 3) [40]. A recent human study reported that the T1 and T2 relaxation times were prolonged within 3 months after chemotherapy, suggesting myocardial edema and inflammation, while an abnormal T1 relaxation time and GLS were observed at later time (>12 months), indicating myocardial fibrosis and ventricular deformation [42]. These findings were consistent with the pathological changes in cardiotoxicity that were identified in animal studies.

T1 mapping and ECV quantification provide a non-invasive method to evaluate myocardial fibrosis, which is characterized by extracellular space expansion and collagen deposition. Increases in native T1 and ECV values can be used to assess diffuse myocardial fibrosis that cannot be identified using LGE [100]. A study of subclinical anthracycline cardiotoxicity suggested that increased native T1 and ECV values, rather than LVEF, were the early markers of ventricular remodeling, and were correlated with chemotherapy dose and wall thickness/height ratios (one of the signs of myocardial atrophy) [101]. On the other hand, decreased native T1 values 48 h after initiation of chemotherapy were observed in patients treated with anthracycline who developed cardiotoxicity upon completion of the therapy, suggesting that native T1 might be useful for risk stratification of cardiotoxicity at a very early stage [91].

ECV refers to the volume of extracellular matrix as a percentage of the total volume of myocardial tissue, which can be calculated using pre- and post-contrast T1 mapping with good reproducibility. ECV is mainly affected by the interstitial space and myocellular volume or mass [102], and has been proven to correlate well with the histological collagen volume fraction and extracellular space [103,104]. Several studies have demonstrated increased ECV in patients [101,105,106,107] and animal models [40,108,109] treated with anthracycline, which may occur as early as 3 months after chemotherapy [107]. An elevated ECV was also found to be associated with an increased risk of LV systolic dysfunction and adverse outcomes of heart failure [105,110].

To sum up, mapping techniques can non-invasively identify subtle changes in myocardial tissue, and provide promising imaging biomarkers for the early detection and prognosis of cardiotoxicity associated with cancer therapies, but they were included in the expert consensus for the clinical management of cardiotoxicity. Moreover, myocardial T1/T2 mapping and ECV values can be affected by various technical and subject-related factors. The current expert consensus for myocardial parametric mapping techniques recommends local reference ranges for clinical interpretation [111].

## 4. Future Directions

CMR has been involved in a number of randomized clinical trials for the assessment of cardiotoxicity in the most recent decade, and was used either as an outcome measure in the evaluation of cancer therapies, or as a diagnostic test for early management of cardiotoxicity. We identified around 20 ongoing or newly completed registered clinical trials involving CMR for cardiotoxicity. Apart from conventional structural and functional evaluation, parametric mapping (Clinicaltrials.gov: NCT03301389, NCT04461223, NCT04486573, NCT01671696, NCT04896242, NCT04036045, and NCT05349058), LV myocardial strain (NCT04486573, NCT05271162, and NCT05349058), RV free wall strain (NCT05349058), two-dimensional flow imaging (NCT04896242), myocardial diffusion weighted imaging (NCT02274480), and hyperpolarized carbon 13-based magnetic resonance spectroscopic imaging (NCT04044872 and NCT03685175) are also involved in the recruiting clinical trials. Moreover, some trials aim to compare the diagnostic value of CMR with other imaging modalities such as CT (NCT04880317) and PET/MRI (NCT04555642). The cancer therapies assessed by these ongoing clinical trials have expanded from chemotherapy to radiotherapy, immunotherapy, and targeted therapy.

With the increasing attention paid to the value of CMR in the clinical management of cardiotoxicity, novel biomarkers reflecting the pathological process of cardiotoxicity are expected to provide new insights into the early diagnosis and prognosis of cardiotoxicity. Emerging CMR techniques such as magnetic resonance spectroscopy (MRS), diffusion weighted imaging (DWI), and diffusion tensor imaging (DTI) may provide additional insights into the metabolic status and the organization and integrity of microstructural components in cardiotoxicity.

Phosphorus MRS (^31^P-MRS) can be used to detect resonance peaks of seven metabolites, among which the ratio of phosphocreatine (PCr) to adenosine triphosphate (ATP) is the most important parameter for analyzing myocardial energy status [112]. The PCr/ATP ratio decreases in a variety of cardiac pathological conditions due to an altered energy metabolism, and the magnitude of the decrease is related to the severity of the cardiac dysfunction [113,114]. Energetic impairment predated contractile dysfunction in a mouse model of cardiotoxicity [115]. In a recent pilot study, however, no clear link between the changes in the PCr/ATP ratio quantified using ^31^P-MRS and anthracycline therapy was found in breast cancer patients [116]. Further studies are needed to investigate the utility of ^31^P-MRS in the identification of energetic abnormalities in cardiotoxicity.

Myocardial DWI and DTI have been technically feasible for patients with cardiomyopathy. The value of DWI as an alternative to native T1 and ECV for the detection and quantification of myocardial fibrosis has been validated in patients with hypertrophic cardiomyopathy [117,118]. While one clinical trial aiming to investigate the value of DWI as a biomarker for the detection of cardiotoxicity has been completed according ClinicalTrial.gov, no results have been published yet. DTI technique can be used to characterize the movement direction and amplitude of water in myocardial fibers at the microscopic level. A recent study showed an acute decrease in fractional anisotropy, the secondary eigenvector angle, and proportions of right-handed myocytes assessed using DTI in acute myocardial infarction, with fractional anisotropy being an independent predictor of LVEF at 3 months [119]. Although there has been no clinical data on myocardial DTI in cancer patients yet, it is worth considering whether DTI may provide a new option for the early detection and prognosis of cardiotoxicity.

Despite the superior qualities of CMR in the assessment of myocardial injury, its application in clinical practice is limited by its relatively high technical requirements and low availability (Table 2), thus the involvement of CMR in clinical situations varies with institutions. However, the tissue characterization property of CMR makes it the most promising non-invasive tool for the early detection of cardiotoxicity. The 2020 SCMR position paper recommended CMR as the first-line imaging technique for several cardiovascular diseases, with T1/T2 mapping involved in the diagnostic criteria of specific diseases, such as myocarditis and cardiac amyloidosis [120]. For cancer-related cardiotoxicity, however, further clinical trials are needed to determine when and how to incorporate CMR for early diagnosis and clinical management of cardiotoxicity. For institutions with well-developed infrastructures for multiparametric CMR, a consecutive imaging strategy assessing the baseline, short-term, and long-term cardiovascular alterations is recommended. T1, T2, and ECV mapping of multiple short-axis slices covering the entire left ventricle, in addition to conventional cine and LGE imaging, are particularly recommended for the evaluation of cardiotoxicity in future clinical trials. We also recommend calculating the global longitudinal strain and circumferential strain for both the left and right ventricles based on cine images. DWI and DTI of the LV myocardium can be incorporated in the protocol for institutions with abundant experience. However, validation studies at a large scale and standardized post-processing tools are still needed for cardiac DWI and DTI techniques before they can serve as potential biomarkers for the clinical management of cardiotoxicity.

## 5. Conclusions

The development and application of cancer therapies have resulted in a higher survival rate with inevitable side effects, the most serious of which is cardiotoxicity. Early diagnosis and treatment of cardiotoxicity are of great significance in the prognoses of patients. Existing studies suggest that myocardial strain and mapping technology based on cardiovascular magnetic resonance imaging are promising for the early diagnosis of cardiotoxicity before the left ventricular ejection fraction deteriorates. The ongoing clinical trials involving strain analysis and parametric mapping assessment may provide substantial data regarding the normal ranges and cut-off values for abnormalities for the novel imaging biomarkers. In addition, the introduction of other techniques, including magnetic resonance spectroscopic imaging, myocardial diffusion weighted imaging, and diffusion tensor imaging, may help to elucidate the underlying pathophysiological mechanism, and thus are worthy of further investigation in future studies.

## Figures and Tables

**Table 1:** Diagnostic criteria for cardiotoxicity.

Society	Diagnostic Criteria of Cardiotoxicity	Year of Publication
**ASE/EACVI**	Decrease in LVEF of >10%, to LVEF < 53% Relative drop in GLS > 15% from baseline suggests subclinical LV dysfunction	2014 [14]
**ESC**	Decrease in LVEF of >10% from baseline, to LVEF < 50% Decrease in GLS of >15% from baseline may suggest risk of cardiotoxicity	2016 [7]
**ESMO**	LVEF drop by ≥10–15%, or to <50% Symptomatic heart failure regardless of LVEF	2020 [13]
**IC-OS**	For asymptomatic patients: Mild: LVEF ≥ 50% and new relative decrease in GLS by >15% from baseline, and/or new rise in cardiac biomarkers (cardiac troponin I/T > 99th percentile, BNP > 35 pg/mL, NT-proBNP ≥ 125 pg/mL). New reduction in LVEF by ≥10% or <10%, to absolute 40% < LVEF < 50%, and new relative decrease in GLS by >15% from baseline, and/or new rise in cardiac biomarkers. Severe: new LVEF reduction to <40%. For symptomatic patients: mild heart failure symptoms or more.	2021 [15]

ASE = American Society of Echocardiography; EACVI = European Association of Cardiovascular Imaging; ESC = European Society of Cardiology; ESMO = European Society of Cardiology; IC-OS = International Cardio-Oncology Society; LVEF = left ventricular ejection fraction; GLS = global longitudinal strain; BNP = B-type natriuretic peptide; NT-proBNP = N-terminal pro-BNP.

**Table 2:** Advantages and limitations of different imaging techniques in the diagnosis of cardiotoxicity.

Imaging Techniques	Monitoring Index or Characteristic	Advantages	Limitations
MUGA	LVEF	Reproducibility	Radiation exposure Limited morphological and functional information of other cardiac chambers and extra-cardiac structures
Echocardiography	LVEF RVEF Strain (GLS, GCS, GRS) LV mass	Wide availability High portability No radiation Morphological and functional information Valvular function Low cost	Suboptimal acoustic window High operator dependency High variability GLS: inter-vendor variability and technical requirements
CMR	LVEF RVEF Strain (GLS, GCS, GRS) LV mass Edema Inflammation Fibrosis	Reproducibility Accuracy No radiation Morphological and functional information Valvular function Tissue characterization	Limited availability High cost Technical requirements Patient adaptation (contraindications for CMR: difficulty in holding breath or lying flat)

MUGA = multi-gated radionuclide angiography; CMR = cardiovascular magnetic resonance; LVEF = left ventricular ejection fraction; RVEF = right ventricular ejection fraction; GLS = global longitudinal strain; GCS = global circumferential strain; GRS = global radial strain.

## Data Availability

Not applicable.
